# Prevention of biliary complications following living donor liver transplantation in Central Asia: a single-centre experience from Kazakhstan

**DOI:** 10.1186/s12893-026-03546-3

**Published:** 2026-02-04

**Authors:** Ayana Mussina, Maksat Doskhanov, Bolatbek Baimakhanov, Shokan Kaniyev, Baglan Askeyev, Susumu Eguchi

**Affiliations:** 1 Department of Hepatobiliary Surgery and Liver Transplantation, The National Scientific Center of Surgery named after. A.N. Syzganov, Almaty, Kazakhstan; 2 Department of Surgery, Nagasaki University Graduate School of Biomedical Sciences, Nagasaki, Japan; 3 S.D. Asfendiyarov Kazakh National Medical University, Almaty, Kazakhstan

**Keywords:** Living donor liver transplantation, Biliary complications, Bile duct anatomy, Biliary stricture, Bile leakage, Central asia

## Abstract

**Background:**

Biliary complications (BCs) remain a frequent and clinically important cause of morbidity after living donor liver transplantation (LDLT), with adverse effects on graft function and long-term survival. However, evidence from Central Asia remains scarce.

**Aim:**

To evaluate the incidence, risk factors, and survival impact of BCs in adult LDLT recipients at a Central Asian centre.

**Methods:**

**This retrospective observational cohort study** included 205 adult recipients who underwent living donor liver transplantation between 2011 and 2024. Biliary complications were defined based on combined clinical, biochemical, and radiological criteria. Patients were stratified according to biliary anatomy and reconstruction technique. Risk factors for biliary complications were evaluated using logistic regression analysis. Overall survival was assessed using the Kaplan–Meier method and compared between groups using the log-rank test.

**Results:**

BCs developed in 50 patients (24.4%). Biliary strictures occurred in 27 patients (54.0%), bile leakage in 15 (30.0%), and combined lesions in 8 (16.0%). Multivariate analysis identified male sex (odds ratio [OR] 2.11, 95% confidence interval [CI] 1.01–4.39; *p* = 0.045) and multiple bile ducts (OR 2.92, 95% CI 1.33–6.39; *p* = 0.008) as independent predictors of BCs. Prolonged cold ischaemia time was significant on univariate analysis but not after adjustment. Overall survival at 1, 3, and 5 years in the entire cohort was 85.7%, 81.0%, and 78.0%, respectively. Patients with BCs demonstrated reduced long-term survival compared with the overall transplant cohort, with survival rates of 93.3%, 78.3%, and 73.3% at the corresponding time points.

**Conclusion:**

In this Central Asian LDLT cohort, multiple bile ducts and male sex were independent risk factors for biliary complications, which were associated with poorer long-term survival. Careful preoperative biliary evaluation, preservation of ductal blood supply, and tailored reconstruction techniques are critical to reducing biliary morbidity and improving outcomes following LDLT.

**Supplementary Information:**

The online version contains supplementary material available at 10.1186/s12893-026-03546-3.

## Introduction

Living donor liver transplantation (LDLT) is a life-saving procedure for patients with end-stage liver disease and acute liver failure. Despite advances in surgical techniques and immunosuppressive management, biliary complications (BCs) remain among the most frequent and clinically relevant adverse events, with reported incidences ranging from 11% to 40%. These complications impair graft function; reduce patient survival, and compromise quality of life, frequently necessitating repeated interventions or retransplantation [1, 2].

The occurrence of BCs is multifactorial. Surgical factors include multiple bile ducts, short ductal stumps, and impaired arterial perfusion, whereas non-surgical mechanisms involve ischemia–reperfusion injury, hypoperfusion, and immunological processes [3, 4]. Compared with deceased donor liver transplantation, LDLT is associated with a higher risk of BCs, as demonstrated in meta-analyses [5]. However, the protective role of splint drainage and the most effective strategies for preventing BCs remain controversial [6–8].

Although these issues are well documented internationally, evidence from Central Asia is limited. To our knowledge, this study represents the first comprehensive analysis of BCs following LDLT in this region. In Kazakhstan, where LDLT has been increasingly performed, particularly at the Syzganov National Scientific Center of Surgery, the incidence, risk factors, and long-term outcomes of BCs have not been systematically evaluated. Clarifying the influence of graft anatomy, including multiple bile ducts, and technical factors such as splint drainage may provide region-specific insights into patient prognosis.

The aim of this study was to evaluate risk factors for biliary complications and to analyze surgical strategies for biliary reconstruction following living donor liver transplantation in a Central Asian cohort, for which published data remain limited. It was assumed that risk factors for biliary complications and clinical outcomes previously reported in international studies may also be reproducible in the Central Asian population, and that analysis of regional experience would help to better characterize local clinical practice and treatment outcomes.

## Materials and methods

This retrospective cohort study included adult patients (≥ 18 years) who underwent living donor liver transplantation (LDLT) at the National Scientific Center of Surgery named after A.N. Syzganov between 2011 and 2024. Patients who underwent deceased donor liver transplantation or pediatric liver transplantation were excluded. Additional exclusion criteria included incomplete clinical or follow-up data.

Participants were identified retrospectively from the institutional liver transplantation database and electronic medical records. All consecutive eligible patients meeting the inclusion criteria during the study period were included to minimize selection bias.

Postoperative follow-up was performed according to institutional protocols and included regular outpatient visits, laboratory testing, and imaging studies as clinically indicated. Follow-up data were obtained from medical records, with biliary outcomes and overall survival assessed throughout the follow-up period.

**Fig. 1:**
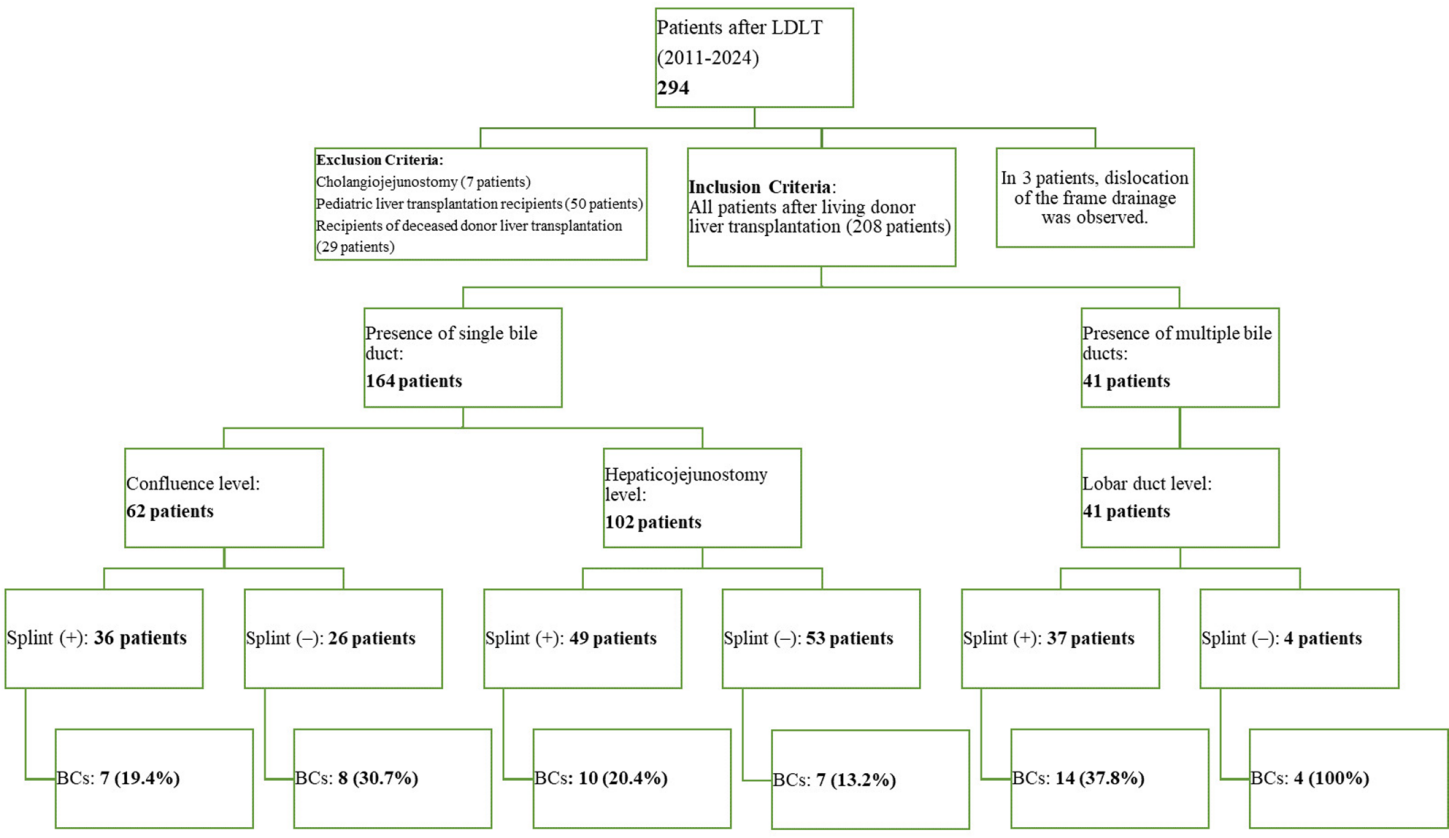
Study design and patient selection flowchart

Biliary reconstruction was performed according to graft bile duct anatomy and duct diameter (Fig. 1).

Figure 1. Study design of the single-centre cohort of adult living donor liver transplantation (LDLT) recipients from 2011 to 2024.

The distribution of biliary reconstruction techniques was as follows:

1. *Single-duct duct-to-duct anastomosis* was performed in 164 patients (80.0%):
  - at the confluence level — 62 patients (37.8%);
  - at the hepaticocholedochus level — 102 patients (62.2%);
2. *Multiple bile ducts requiring ductoplasty at the lobar level* were present in 41 patients (20.0%);
3. *Combined or other reconstruction techniques* were not used.

Detailed surgical characteristics are presented in Table 2.

**Fig. 2:**
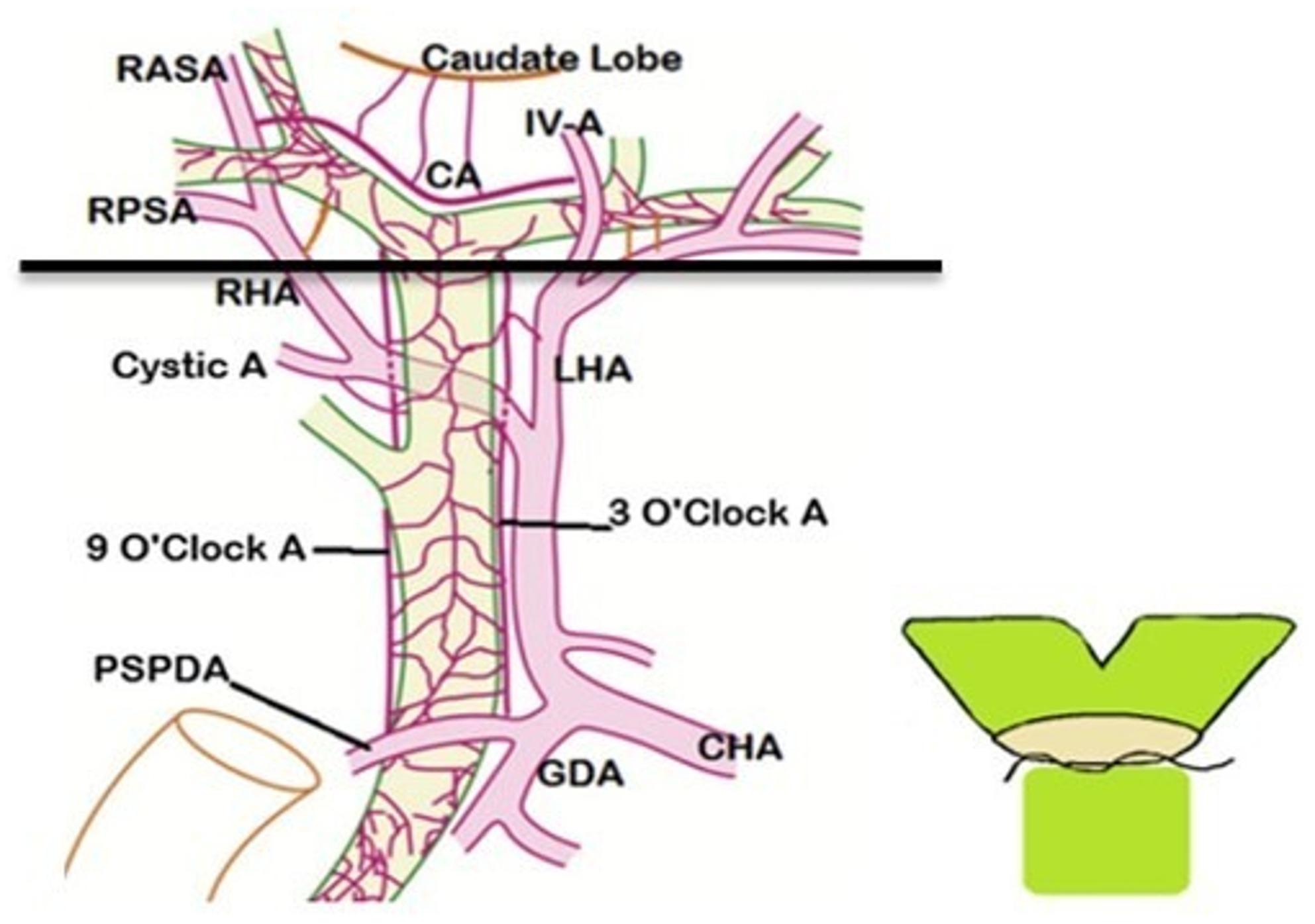
Schematic illustration of biliary reconstruction strategies according to the level of anastomosis (confluence-level vs. hepaticocholedochal-level)

**Fig. 3:**
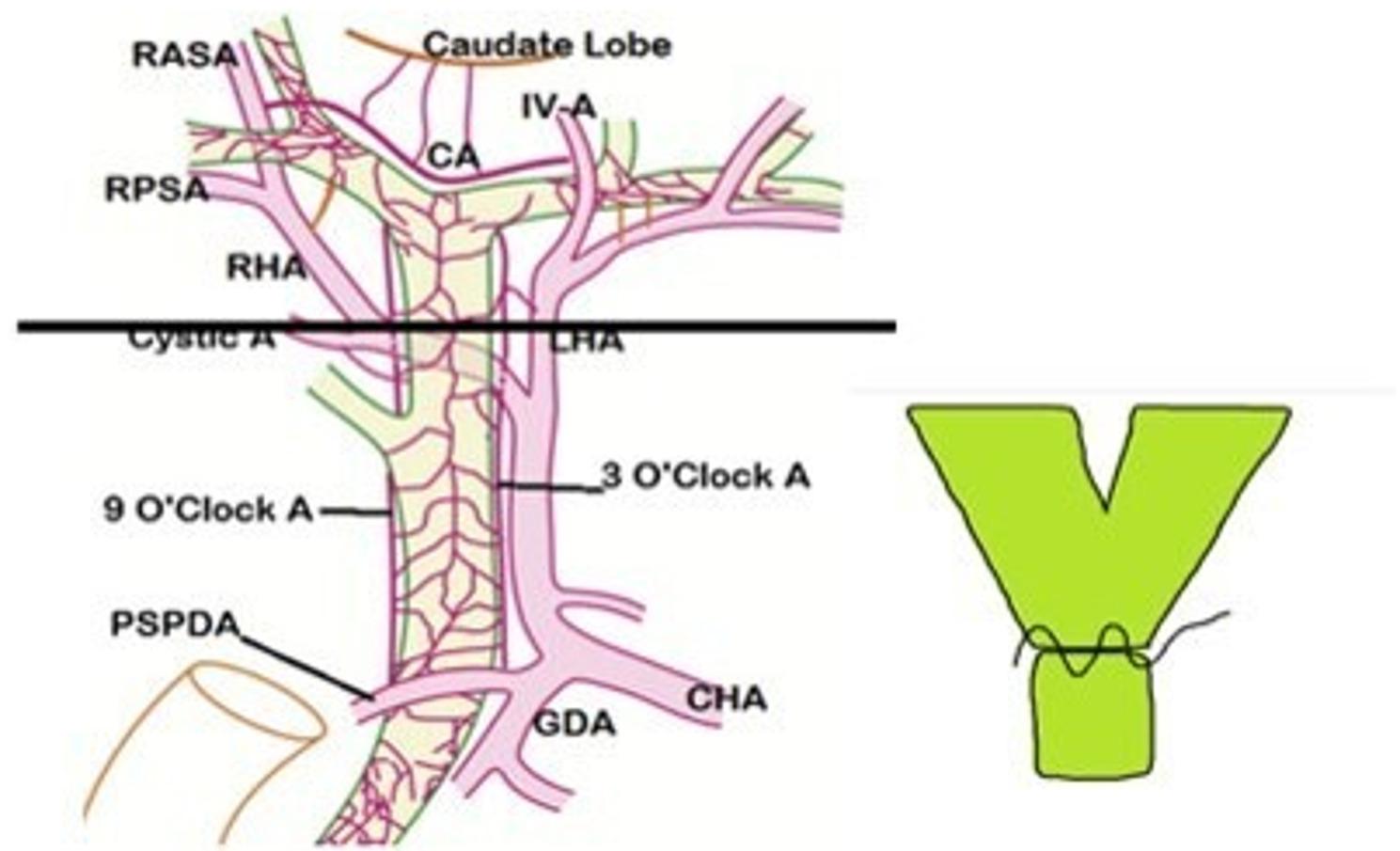
Schematic representation of ductoplasty technique for grafts with multiple bile ducts

Intraoperatively, the level of biliary reconstruction was classified according to the anatomical position of bile duct division relative to the hepatic duct confluence (Figs. 2 and 3). A confluence-level anastomosis was defined when bile duct transection was performed at or within 3–5 mm of the junction of the right and left hepatic ducts, resulting in a short common hepatic duct stump (Fig. 2). A hepaticocholedochal-level anastomosis was defined when division occurred distal to the confluence along the common hepatic duct, allowing reconstruction using a single duct with sufficient length (Fig. 3).

In cases with multiple bile duct orifices, ductoplasty was undertaken whenever technically feasible. Ductoplasty involved longitudinal incision of adjacent duct walls followed by side-to-side unification using interrupted 6 − 0 absorbable monofilament sutures to create a single biliary orifice for subsequent anastomosis. When unification was not feasible due to wide separation or marked size discrepancy, separate duct-to-duct anastomoses were performed. The reconstruction technique was selected based on duct anatomy, duct diameter, inter-duct distance, and recipient bile duct quality.

**Fig. 4:**
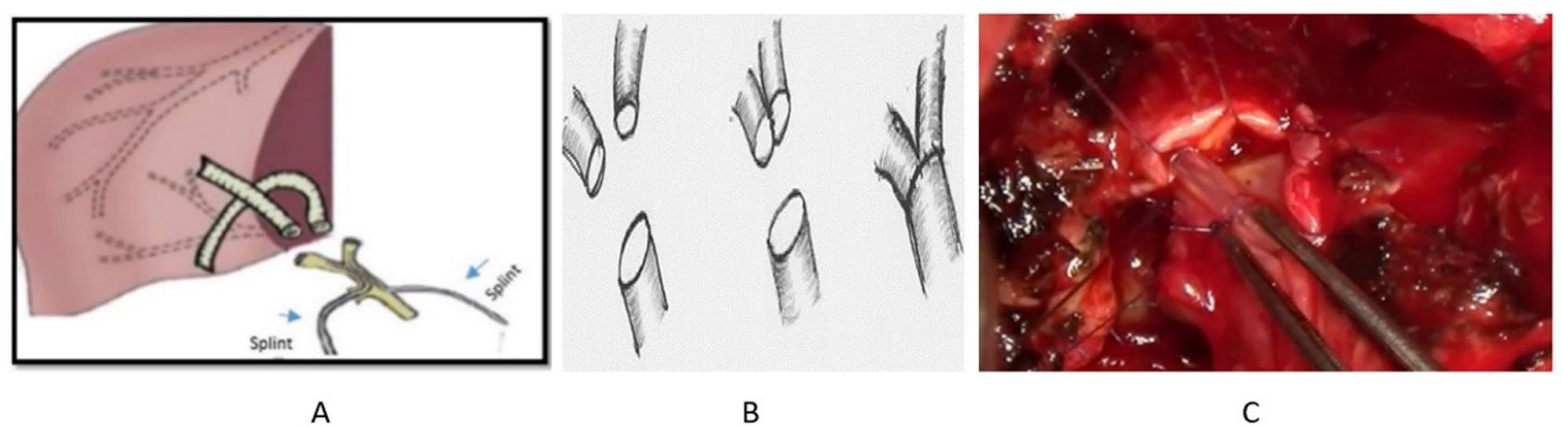
Biliary reconstruction strategies for grafts with multiple bile ducts. **A** Double duct-to-duct biliobiliary anastomosis in a graft with two bile ducts with internal splint drainage. **B** Ductoplasty for multiple bile ducts using a side-to-side unification technique to form a single biliary lumen. **C** Intraoperative photograph showing completed ductoplasty with formation of a single common biliary orifice before biliary anastomosis.

In grafts with multiple bile ducts, biliary reconstruction was tailored according to duct anatomy. When two distinct duct orifices were present, a double duct-to-duct biliobiliary anastomosis with internal splint drainage was performed (Fig. 4A). When multiple ducts were closely positioned, ductoplasty was performed by longitudinal incision of adjacent ductal walls followed by side-to-side unification to create a single biliary lumen suitable for subsequent biliary anastomosis (Fig. 4B). The final intraoperative appearance of completed ductoplasty with a single common biliary orifice is shown in (Fig. 4C).

### Definition of biliary complications

BCs were identified using combined clinical, laboratory, and radiological criteria. Clinical suspicion was based on obstructive jaundice or biliary leakage. Laboratory abnormalities included elevated direct bilirubin and transaminases. Magnetic resonance cholangiopancreatography (MRCP) was used to assess biliary strictures and bile flow, while abdominal ultrasound and drain output were used to confirm bile leakage and biloma formation.

Bile leakage was defined by the presence of bilious drain output, supportive laboratory findings, and radiological evidence of leakage or biloma requiring interventional or surgical management, in accordance with definitions used in previous LDLT studies. For biliary strictures, the date of radiological confirmation MRCP or cholangiography was considered the time of occurrence; biochemical abnormalities or the timing of intervention were not used for this definition [9, 10]. 

### Outcomes

The primary endpoint was the occurrence of biliary complications. Secondary endpoints included patient and graft survival, as well as associations between BCs and anatomical or technical factors, including number of bile ducts, level of anastomosis, and use of splint drainage.

Several measures were taken to minimize potential sources of bias. To reduce selection bias, all consecutive adult patients undergoing living donor liver transplantation during the study period were included according to predefined inclusion and exclusion criteria. Information bias was minimized by using standardized data sources, including electronic medical records, operative reports, and the institutional liver transplantation database.

### Statistical analysis

Descriptive statistics were used to summarize baseline characteristics. Continuous variables are presented as mean ± standard deviation or median with interquartile range, as appropriate, while categorical variables are reported as counts and percentages. Group comparisons were performed using appropriate parametric or non-parametric tests.

Univariate analyses were conducted to identify factors associated with biliary complications. Variables with *p* < 0.10 in univariate analysis were entered into a multivariable logistic regression model to identify independent predictors of biliary complications and to control for potential confounding. Given the total number of biliary complication events (*n* = 50), the number of variables included in the multivariable model was limited to ensure model stability. Collinearity among covariates was assessed prior to multivariable analysis, and no significant multicollinearity was detected.

Subgroup analyses were performed according to the type of biliary complication (bile leakage and biliary stricture), biliary anatomy, and level of biliary reconstruction. Formal statistical tests for interaction were not performed.

Missing data were minimal and were handled using complete-case analysis; no imputation methods were applied. There was no loss to follow-up during the study period, and all included patients were available for outcome analysis. Sensitivity analyses were not performed.

Results are reported as odds ratios (ORs) with 95% confidence intervals (CIs).

The sample size was based on all available cases (*n* = 205). Assuming an incidence of biliary complications of approximately 25%, the study had > 80% statistical power at a two-sided α level of 0.05 to detect a clinically meaningful difference in survival between patients with and without biliary complications. Statistical analyses were performed using SAS version 9.4 (SAS Institute, Cary, NC), and a two-tailed *p*-value ≤ 0.05 was considered statistically significant.

## Results

A total of 205 liver transplant recipients were included. Of these, 155 recipients (75.6%) did not develop BCs, whereas 50 (24.4%) experienced BCs.

### Baseline characteristics

The mean age of the cohort was 44.0 ± 11.1 years, with no significant difference between patients with and without BCs (44.1 ± 12.1 vs. 43.9 ± 10.8 years, *p* = 0.81). Males comprised 37.6% of the cohort and were significantly more frequent among patients with BCs than among those without BCs (52.0% vs. 32.9%, *p* = 0.020). The mean BMI was 23.5 ± 3.9, and the mean MELD score was 17.6 ± 6.3. Most patients were classified as Child–Pugh B (60.5%), followed by Child–Pugh C (35.1%) and Child–Pugh A (4.4%). Hepatitis B virus + hepatitis D virus infection was the most common aetiology (50.2%). No other baseline variables differed significantly between groups (Table 1).

**Table 1:** Baseline characteristics of LDLT recipients (*n* = 205)

Variable	Total (*n* = 205)	Without BCs (*n* = 155)	With BCs (*n* = 50)	*p*-value
Recipient characteristics				
Age (years), mean ± SD	44.0 ± 11.1	43.9 ± 10.8	44.1 ± 12.1	0.81
Sex				
Male, n (%)	77 (37.6%)	51 (32.9%)	26 (52.0%)	0.02
Female, n (%)	128 (62.4%)	104 (67.1%)	24 (48.0%)	
BMI, mean ± SD	23.5 ± 3.9	23.5 ± 4.0	23.4 ± 3.7	0.96
Aetiology of liver disease				
HBV, n (%)	32 (15.6%)	20 (12.9%)	12 (24.0%)	0.26
HBV + HDV, n (%)	103 (50.2%)	80(51.6%)	23 (46.0%)	
HCV, n (%)	23 (11.2%)	20 (12.9%)	3 (6.0%)	
Cryptogenic, n (%)	7 (3.4%)	5 (3.2%)	2 (4.0%)	
Primary biliary cirrhosis, n (%)	24 (11.7%)	20 (12.9%)	4 (8.0%)	
Primary sclerosing cholangitis, n (%)	7 (3.4%)	5 (3.2%)	2 (4.0%)	
Autoimmune hepatitis, n (%)	8 (3.9%)	4 (2.6%)	4 (8.9%)	
Budd–Chiari syndrome, n (%)	1 (0.5%)	1 (0.6%)	0	
MELD score, mean ± SD	17.6 ± 6.3	17.5 ± 6.3	17.9 ± 6.4	0.76
Child–Pugh class				
A, n (%)	9 (4.4%)	7 (4.5%)	2 (4.0%)	0.32
B, n (%)	124 (60.5%)	98(63.2%)	26 (52.0%)	
C, n (%)	72 (35.1%)	50 (32.3%)	22 (44.0%)	
Donor characteristics				
Age	29.0 ± 8.4	28.6 ± 8.6	30.3 ± 7.9	0.10
Sex				
Male, n (%)	118 (57.6%)	93 (60.0%)	25 (50.0%)	0.25
Female, n (%)	87 (42.4%)	62(40.0%)	25 (50.0%)	
Graft type				
Right lobe, n (%)	181 (88.3%)	133 (85.8%)	48 (96.0%)	0.15

*BCs* biliary complications, *BMI* body mass index, *HBV* hepatitis B virus, *HDV* hepatitis D virus, *HCV* hepatitis C virus, *MELD* Model for End-Stage Liver Disease, *SD* standard deviation

### Surgical characteristics

The mean donor age was 29.0 ± 8.4 years, with male donors accounting for 57.6%. Right lobe grafts were used in 88.3% of patients. Operative time tended to be longer in patients with BCs, although the difference was not statistically significant (769.3 ± 198.4 vs. 717.4 ± 181.8 min, *p* = 0.09). Cold ischaemia time was significantly prolonged in patients with BCs (81.9 ± 60.1 vs. 64.5 ± 59.0 min, *p* = 0.02); however, this association was not retained in multivariate analysis. Warm ischaemia time and splint drainage use did not differ significantly between groups (Table 2).

**Table 2:** Surgical characteristics of LDLT recipients

Variable	Total (*n* = 205)	Without BCs (*n* = 155)	With BCs (*n* = 50)	*p*-value
Operative time	729.0 ± 186.8	717.4 ± 181.8	769.3 ± 198.4	0.09
Cold ischaemia	69.0 ± 59.6	64.5 ± 59.0	81.9 ± 60.1	0.02
Warm ischaemia	36.4 ± 16.7	36.7 ± 17.1	35.5 ± 15.5	0.37
Anastomosis type				
Duct-to-duct, n (%)	196 (95.6%)	148 (95.5%)	48 (96.0%)	> 0.99
Roux-en-Y Hepaticojejunostomy, n (%)	9 (4.4%)	7 (4.5%)	2 (4.0%)	
Splint use				
Yes, n (%)	118 (57.6%)	85 (54.8%)	33 (66.0%)	0.19

*BCs* biliary complications, *LDLT* living donor liver transplantation, *SD* standard deviation

### Biliary complications

Among the 50 patients with BCs, biliary strictures occurred in 27 patients (54.0%), bile leakage in 15 (30.0%), and combined stricture and leakage in 8 (16.0%) (Table 3).

**Table 3:** Types of biliary complications (*n* = 50)

Complication Type	Number of Cases
Biliary stricture, n (%)	27 (54.0%)
Bile leakage, n (%)	15 (30.0%)
Stricture+Bile leakage, n (%)	8 (16.0%)

### Risk factors

Multivariate logistic regression identified multiple bile ducts (OR 2.92; 95% CI: 1.33–6.39; *p* = 0.008) and male sex (OR 2.11; 95% CI: 1.01–4.39; *p* = 0.045) as independent predictors of BCs. Donor age, graft type, splint drainage, operative time, and cold ischaemia time > 90 min were not significant in the adjusted model (Table 4).

**Table 4:** Multivariate analysis of risk factors for biliary complications

Risk factor	Odds ratio (95% confidence interval)	*p*-value
Recipient sex	2.11 (1.01–4.39)	0.045
Donor Age	1.02 (0.98–1.06)	0.34
Multiple duct	2.92 (1.33–6.39)	0.008
Splint use	2.01 (0.84–4.8)	0.11
Operation duration	1.0 (1.0–1.0)	0.082
Cold ischaemia	1.0 (1.0–1.01)	0.18

BCs were further classified as early or late. Early BCs were predominantly bile leaks, whereas late BCs were mainly biliary strictures. A detailed comparison of early and late BCs is provided in Table 5.

**Table 5:** Early and late biliary complications

Type of complication	Early BCs (*n* = 28)	Late BCs (*n* = 22)	Z statistic	OR	95% CI	*p*-value
Leaks, *n* (%)	18 (64.3%)	4 (18.2%)	–	8.6 γ	[2.3; 32.2]	0.0015*
Strictures, *n* (%)	7 (25.0%)	15(68.2%)	–	0.18 β	[0.05; 0.60]	0.005*
Mixed complications, n (%)	3 (10.7%)	3 (13.6%)	–	0.71 β	[0.14; 3.6]	0.682
Fever > 38 °C, n (%)	16(57.1%)	5 (22.7%)	–	4.8 γ	[1.4; 16.6]	0.013*
Cholangitis, *n* (%)	8 (28.6%)	11 (50.0%)	–	0.37 β	[0.1; 1.2]	0.089
Total bilirubin, µmol/L	56.8 ± 22.1	42.3 ± 19.8	2.44*	–	–	0.018*
GGT, U/L	214 ± 92	287 ± 110	2.58*	–	–	0.013*
ERCP, *n* (%)	19 (67.9%)	10 (45.5%)	–	2.7 γ	[0.8; 8.6]	0.084
PTBD, *n* (%)	6 (21.4%)	7 (31.8%)	–	0.62 β	[0.2; 2.2]	0.465
Number of procedures, mean ± SD	1.7 ± 0.9	2.4 ± 1.2	2.38*	–	–	0.021*
Treatment duration, days	22.6 ± 8.1	36.2 ± 9.7	5.46*	–	–	< 0.001*

*BCs* biliary complications, *OR* odds ratio, *CI* confidence interval, *ERCP* endoscopic retrograde cholangiopancreatography, *PTBD* percutaneous transhepatic biliary drainage, *GGT* gamma-glutamyl transferase, *SD* standard deviation

OR: odds ratio; α: OR = 1 indicates equal odds between groups; β: OR < 1 indicates an inverse association; γ: OR > 1 indicates a direct association

*z-test statistical significance; *p*-value ≤ 0.05 was considered statistically significant

Early and late biliary complications differed in their clinical presentation, biochemical profile, and management strategies (Table 5). Early BCs were predominantly bile leaks and were more often associated with systemic inflammatory manifestations, including fever, as well as higher total bilirubin levels. In contrast, late BCs were mainly biliary strictures, characterised by higher GGT levels, a tendency toward cholangitis, and a greater need for repeated therapeutic interventions, resulting in a significantly longer duration of treatment.

### Survival outcomes

**Fig. 5:**
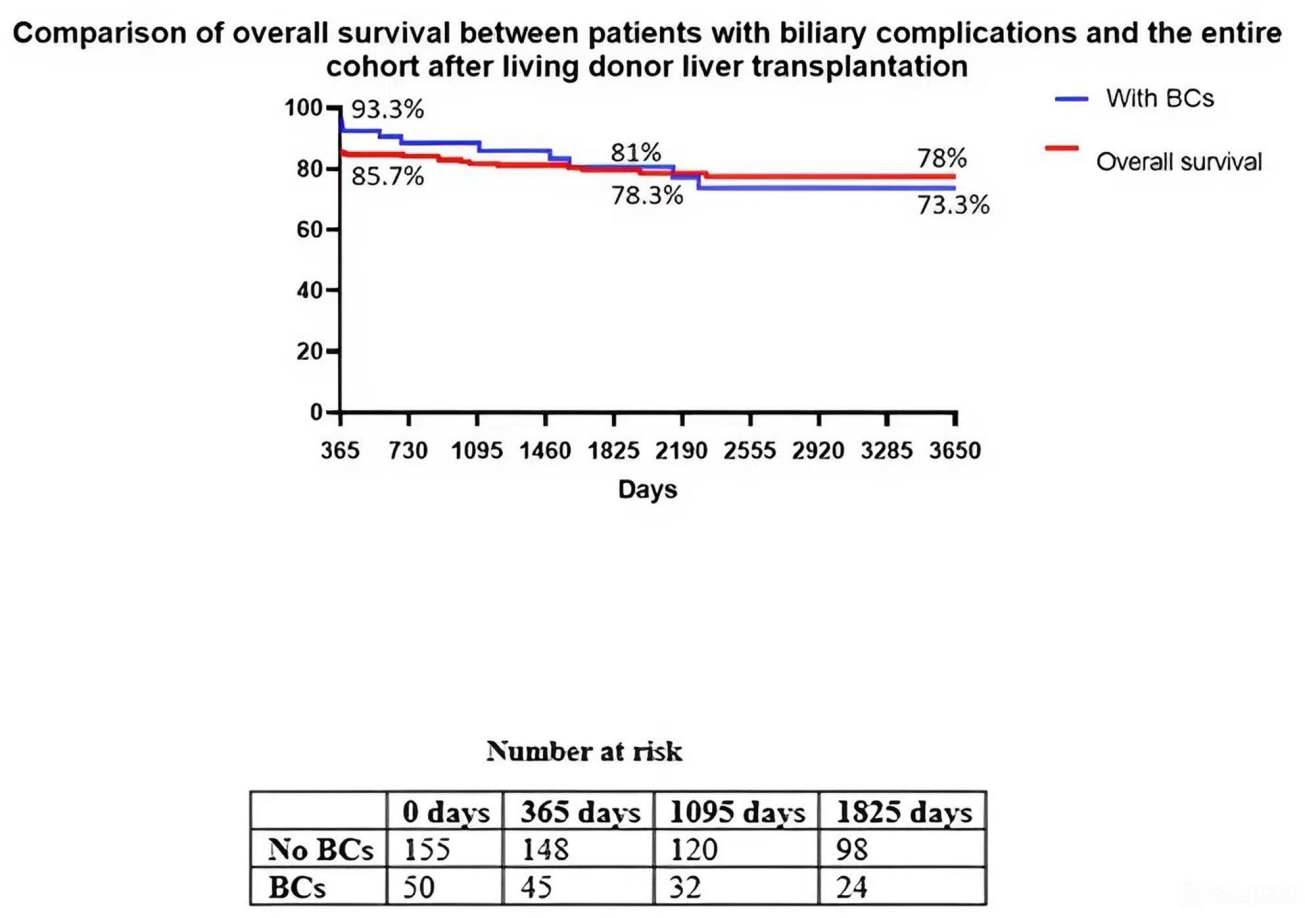
Comparison of overall survival between patients with biliary complications and the entire cohort after living donor liver transplantation

Overall survival after living donor liver transplantation was analysed using the Kaplan–Meier method (Fig. 5). Patients with biliary complications demonstrated consistently lower survival compared with patients without biliary complications. At 1, 3, and 5 years, overall survival in the entire cohort was 85.7%, 81.0%, and 78.0%, respectively, whereas survival among patients with biliary complications was 93.3%, 78.3%, and 73.3%. The number of patients at risk decreased progressively over time in both groups, with a more pronounced decline in the biliary complications group.

Figure 5. Comparison of overall survival between patients with biliary complications and the entire cohort after living donor liver transplantation. Kaplan–Meier curves show survival in patients with biliary complications (blue line) and in the overall transplant cohort (red line). The table beneath the graph presents the number of patients at risk at predefined time points.

On multivariate analysis, multiple bile ducts (OR 2.92; 95% CI: 1.33–6.39; *p* = 0.008) and male sex (OR 2.11; 95% CI: 1.01–4.39; *p* = 0.045) remained independent predictors of BCs. All statistical results were cross-checked for consistency across the text, tables, and figures.

## Discussion

In this retrospective single-centre study from Kazakhstan, BCs occurred in 24.4% of adult patients after LDLT. This incidence lies within the internationally reported range of 13–40% and is comparable with large series from high-volume centres, including the study, which reported a 19% incidence among 2,812 LDLT recipients. These findings confirm that BCs remain a major clinical challenge following LDLT despite continued advances in surgical technique and perioperative care [11].

Biliary strictures were the most frequent complication, accounting for 54% of BCs, followed by bile leakage (30%) and combined lesions (16%). This distribution is consistent with previous studies demonstrating that anastomotic strictures represent the predominant form of biliary morbidity after LDLT [12]. Such complications are technically demanding to manage, are frequently related to ischemic injury, and often require repeated endoscopic or percutaneous interventions, thereby substantially increasing the burden of care [13].

We also observed that the level of biliary anastomosis influenced the occurrence of biliary complications. Among patients who underwent duct-to-duct anastomosis, biliary complications occurred in 15 of 62 recipients with a confluence-level anastomosis (24.2%) and in 17 of 102 recipients with a hepaticocholedochus-level anastomosis (16.7%). These findings suggest that anastomoses performed at the confluence level may be associated with a higher risk of biliary complications, possibly due to a shorter ductal stump and compromised blood supply. However, the level of biliary anastomosis did not emerge as an independent predictor in multivariate analysis.

Multivariate analysis identified male sex and the presence of multiple bile ducts as independent risk factors for BCs. The association between complex biliary anatomy and increased biliary morbidity is well established and reflects the technical challenges of reconstruction, including multiple anastomoses or ductoplasty and heightened susceptibility to ischemic injury [14]. In the Central Asian setting, where donor availability may be limited, the use of grafts with complex biliary anatomy is sometimes unavoidable, underscoring the importance of meticulous preoperative biliary mapping and individualized reconstruction strategies [15].

Male sex was independently associated with a higher risk of BCs. Although the underlying mechanisms remain unclear, similar associations have been reported in other LDLT cohorts and may reflect indirect factors, such as donor–recipient size mismatch, anatomical variation, or perioperative haemodynamic conditions, rather than a direct biological effect [16].

Cold ischemia time was associated with BCs in univariate analysis, supporting evidence that ischemia–reperfusion injury contributes to cholangiocyte damage and subsequent stricture formation. However, this association did not persist in multivariate analysis, suggesting confounding by anatomical or technical variables [17]. Nonetheless, minimising ischemia time remains a fundamental principle of LDLT, particularly in geographically large regions such as Central Asia, where logistical challenges may prolong organ transport and preservation times [18].

Arterial complications are recognised contributors to ischemic cholangiopathy. In this cohort, arterial events were uncommon, and only two patients subsequently developed BCs, both of whom achieved favourable outcomes following timely surgical revision. This finding highlights the importance of early detection and prompt management of arterial compromise [19].

The role of internal splint drainage remains controversial. While some studies advocate routine use, others have reported increased risks of obstruction or infection. In this cohort, splint drainage was applied selectively, primarily in grafts with multiple bile ducts. Although splint use was not identified as an independent protective factor, the observation that all patients with multiple bile ducts who did not receive splint drainage developed BCs supports a selective, anatomy-based approach [20].

Survival analysis demonstrated that BCs had a significant adverse effect on long-term outcomes. Recipients with BCs exhibited lower survival than those without complications, consistent with previous reports linking biliary morbidity to reduced survival and impaired quality of life after liver transplantation [21]. In Central Asia, where repeated hospitalisations and complex interventions place considerable strain on healthcare resources, prevention of BCs has important clinical and socioeconomic implications [22]. BCs after LDLT in Central Asia occur at rates comparable to those reported internationally and are strongly influenced by graft biliary anatomy and recipient characteristics. The identification of multiple bile ducts and male sex as independent risk factors emphasises the need for meticulous preoperative planning, individualized biliary reconstruction, and intensified postoperative surveillance in high-risk patients. As LDLT continues to expand across Central Asia, adoption of risk-adapted strategies informed by both international evidence and regional experience may reduce biliary morbidity and improve long-term graft and patient survival [23, 24].

### Limitations

This study has several limitations that should be acknowledged. First, its retrospective single-centre design may limit the generalisability of the findings and precludes causal inference. Second, the relatively small number of patients with biliary complications may have reduced the statistical power to detect modest associations. In addition, potentially relevant variables, including differences in immunosuppressive regimens and the learning curve associated with the development of a living donor liver transplantation programme, were not fully accounted for and may have influenced the observed outcomes. Despite these limitations, the present study provides clinically meaningful insights into anatomical and technical risk factors for biliary complications in the setting of living donor liver transplantation.

## Conclusions

In this single-centre retrospective study, biliary complications after living donor liver transplantation in Central Asia were observed at rates comparable to those reported in international series. The presence of multiple bile ducts and male sex were identified as factors associated with an increased risk of biliary complications. These findings suggest that careful preoperative assessment of biliary anatomy, thoughtful selection of reconstruction techniques, and closer postoperative monitoring may be particularly relevant in recipients at higher risk. In the context of emerging LDLT programmes in Central Asia, incorporation of risk-adapted approaches, informed by both regional experience and existing literature, may help reduce biliary morbidity and support improved long-term graft and patient outcomes. Further prospective, multicentre studies are warranted to validate these observations.

## Supplementary Information

Supplementary Material 1.

## Data Availability

The datasets generated and analysed during this study are available from the corresponding author upon reasonable request.
